# Women's experiences of trauma-informed care for forced migrants: A qualitative interview study

**DOI:** 10.1016/j.heliyon.2024.e28866

**Published:** 2024-03-29

**Authors:** Linda Jolof, Patricia Rocca, Tommy Carlsson

**Affiliations:** a The Red Cross Treatment Center for Persons Affected by War and Torture, Malmö, Sweden; b The Department of Health Sciences, The Swedish Red Cross University, Huddinge, Sweden; c The Department of Women's and Children's Health, Uppsala University, Uppsala, Sweden

**Keywords:** Armed conflicts, Internal displacement, Refugees, Trauma-informed care, Women

## Abstract

**Introduction:**

Forced migration affect the health and wellbeing of millions of women. The aim was to explore experiences of trauma-informed care among women who are forced migrants.

**Methods:**

This was an exploratory qualitative study. Eleven women who had concluded treatment at multidisciplinary trauma centers in Sweden were interviewed, recruited through consecutive sampling. Audio-recorded interviews were transcribed and analyzed with systematic text condensation.

**Results:**

Women dealt with mental and physical manifestations in a challenging psychosocial situation. Various structural and individual barriers were addressed that hindered access to adequate health services. Women appreciated various benefits of the treatment and recalled the care as supportive and compassionate. However, undergoing treatment was considered demanding, requiring significant determination and energy. Participants suggested that peer support could enhance the support.

**Conclusions:**

Migrant women experience a range of health-related burdens and encounter barriers to trauma-informed care. While demanding, treatment has the potential to alleviate symptoms. Health professionals and stakeholders providing trauma-informed care need to ensure that their services are accessible and culturally sensitive towards the unique needs of women. Peer support has the potential to enhance support further, which need further evaluation.

## 1 Introduction

Globally, forced displacement affects the health and wellbeing of millions each year [1]. Persons with experience of armed conflicts, torture, and forced migration are at risk of developing severe health-related consequences, including major depressive disorder and post-traumatic stress disorder [2,3]. A large proportion of forced migrants are women, who are at risk of being exposed to significant gender-based risks and violence before and during their migration journey [[4], [5], [6]]. Gender has a significant influence on the exposure to trauma related to armed conflicts and forced migration, as women encounter unique challenges and consequences when being exposed to such conditions [5]. Indeed, there is evidence that these women are at risk of experiencing a range of mental health burdens [7,8] and somatic illnesses [8,9]. In addition to the significant health-related challenges and consequences before and during the transit [5], refugee women also experience health-related disparities after arrival in the host country. Within the population of resettled and asylum seeking forced migrants, being a woman is associated with a higher risk of developing post-traumatic stress disorder [10] and lower health-related quality of life [11]. Compared to non-migrant populations, refugee women have a higher risk of worse perinatal health outcomes, including postnatal depression, maternal mortality, sexual assault, and unwanted pregnancy [12]. After having arrived in the host country, women who are forced migrants experience structural inequalities and unmet needs of support from health services [9,13]. This calls attention to the need to address global inclusion health [14].

Host countries have an undeniable responsibility to ensure that forced migrants with experience of traumatic events have access to adequate professional support following their arrival [15,16]. Thus, many countries have implemented specialized units providing multidisciplinary trauma-informed care (TIC) for forced migrants with experience of traumatic events. In the literature, TIC is defined as person-centered support of those with experience of physical and/or emotional trauma, based on adequate knowledge, recognition, respect and concern [17]. Based on the available research about the intersecting and unique health-related challenges experienced among women who are forced migrants, gender need to be carefully considered when providing TIC. Referred to as the capacity to bounce back from stressful or traumatic events [18], resilience is suggested to be associated with better mental health among forced migrants [19] and is acknowledged as a fundamental aspect in TIC for women [20]. While resilience is a main theme influencing the health and wellbeing of refugee women, the underrepresentation in research calls attention to the need for more exploratory studies [21].

Previous research has highlighted various challenges that migrant women experience when seeking health services, including in obstetric [22] and primary care [23]. In-depth knowledge regarding these women's experiences of TIC in specialized centers has the potential to enhance the alignment between treatment and the needs women articulate. Qualitative research can provide valuable insights about forced migrants' experiences of receiving professional support within health services in the host country. However, limited inductive and exploratory research has been conducted to understand how clients experience the support received within specialized health services providing multidisciplinary TIC. The aim of this study was to explore experiences of trauma-informed care among women who are forced migrants. Our goal was to explore: (i) how women perceive the treatment activities and support from health professionals, (ii) women's descriptions of the benefits and adverse events of the treatment, and (iii) how they propose that services can be improved to more appropriately address women's needs of support.

## 2 Methods

### 2.1 Design

This was an exploratory study based on interviews with women who have undergone treatment at specialized centers applying TIC for forced migrants resettling in Sweden.

### 2.2 Study context

In Sweden, forced migrants have the right to seek asylum based on a fear of persecution. Cases are handled by the Swedish Migration Agency, which is responsible for arranging housing and financial support for asylum seekers. In Sweden, all forced migrants (including asylum seekers and undocumented migrants) have the right to access healthcare that cannot be deferred. The study was conducted in Sweden and focuses on the six national Swedish Red Cross treatment centers for persons with trauma afflicted by armed conflicts, torture, and/or forced migration. The Swedish Red Cross treatment centers receive referrals from health services, and some centers also accept self-referrals submitted by migrants. The support offered at the centers is free of change. All clients are offered and can decline interpreter services. The centers offer multidisciplinary TIC, in group-based and/or individual therapy sessions. A range of professions are represented at the centers, including psychologists, psychotherapists, physiotherapists, social workers, nurses, and physicians. Forced migrants are offered a range of therapies such as narrative exposure therapy, eye movement desensitization and reprocessing, prolonged exposure, body awareness, yoga, physical activity, psychosocial support, and medical treatment. At the centers, clients typically meet their psychologist and physiotherapist once a week. The treatment can continue over the course of years, with a usual treatment period of approximately one or two years. Individual treatment includes trauma-focused support (e.g., psychotherapy, individualized physiotherapy, and psychosocial support), whereas group-based sessions include physiotherapy together with other forced migrants. The treatment is concluded once the health professionals and the client agree that the goals of their treatment has been fulfilled, or alternatively, when the client is no longer able to participate in the treatment.

### 2.3 Participant recruitment and sample characteristics

Participants were recruited through multi-center sampling. Lists were obtained of women who had concluded their treatment at one of five of the Swedish Red Cross refugee trauma centers. Women were approached in reverse chronological order, starting with the last woman who concluded her treatment at the respective center. To be included, participants needed to be 18 years or older and have concluded their treatment at the center (regardless of the reason for conclusion). Potential participants were contacted via telephone and asked to participate in the study by someone who had not been involved in their treatment, either a member of the research team or an administrator at one of the centers. In total, 11 cisgender women were included in the final sample (center A: n = 1, center B: n = 3, center C: n = 3, center D: n = 1, center E: n = 3). Table 1 presents the sample characteristics. The median time spent in Sweden was 7 years (range: 2–32). Participants originated from nine different countries and their migration statuses ranged from asylum seeker to permanent residence/Swedish citizenship. Participants represented various levels of education and employment statuses.

**Table 1:** Background characteristics of the sample (n = 11 participants).

Variable		n
Age (years)	21–30	2
	31–40	4
	41–50	2
	51–60	1
	61–70	2
Country of birth	Afghanistan	1
	Azerbaijan	1
	Colombia	1
	Iraq	1
	Iran	1
	Kurdistan	2
	Lebanon	2
	Sudan	1
	Syria	1
Legal status	Permanent residence	1
	Swedish citizen	4
	Temporary residence	5
	Asylum seeker	1
Educational level	None	1
	Elementary school	3
	High school or vocational training	2
	University or equivalent	5
Current employment	Yes	6
	No	4
	Retired	1

### 2.4 Data collection

We conducted interviews either face-to-face at the center where they had been treated (n = 6 participants) or digitally via an online video conferencing tool/via telephone (n = 5 participants). Participants were interviewed by one of the first two authors. A semi-structured interview guide (Table 2) was utilized during the interviews, which we developed before conducting interviews. Follow-up, probing, and clarifying questions were asked as needed. A professional interpreter was used in seven of the interviews (carried out in Arabic, Spanish, and Farsi). The interview length ranged between 40 and 60 (median: 50) minutes. All interviews were audio recorded and transcribed verbatim by the interviewers.

**Table 2:** Semi-structured interview guide.

Subject	Main question	Questions
General health	How would you describe your health as it is today?	How is your mental health?
		How is your physical health?
The treatment at the center	What treatment did you attend at the center?	Which professions did you encounter when you were in treatment?
		Did you participate in any individual treatment, and if so, what kind?
		Did you participate in any group-based treatment, and if so, what kind?
		Did you take part in any activity, other than the treatment, at the center?
	How did you experience your treatment?	What did you feel was good or helpful during the treatment?
		What did you feel was less good or helpful during the treatment?
		Was anything lacking during the treatment?
		Was there anything you would have liked more of during the treatment?
Health-related impact of the treatment	What would you say the treatment at the center has meant for your health?	What positive effects on your mental health has the treatment had?
		What positive effects on your physical health has the treatment had?
		What negative effects on your mental health has the treatment had?
		What negative effects on your physical health has the treatment had?
		What have you used, based on what you learned during the treatment?
Care and support for women	What do you think is important for health services to implement, in order to meet women's needs for care and support?	What do you think might prevent women, who are in a similar situation as you were in, from participating in the treatment offered at the center?
		What do you think can facilitate women, who are in a similar situation as you were in, to participate in the treatment at the center?
		How do you think that treatment and other support at the center can be tailored to better suit women's conditions and needs?
		What do you think is important for the staff at the center to consider when they meet women in a similar situation as you were in?
Conclusion of interview	The interviewer gives a summary of what was said during the interview	Is there anything you would like to add?
		Have I misunderstood anything?

### 2.5 Data analysis

The transcribed interviews were analyzed inductively with systematic text condensation, which is a qualitative method for thematic cross-case analysis [24]. The three authors began by reading all transcripts individually, identified preliminary themes, and discussed these until they reached agreement on six preliminary themes. The analysis continued in an iterative process by: (i) coding the material into code groups derived from the preliminary themes, (ii) identifying subgroups in the code groups and coding into these, (iii) producing condensates and identifying an illustrative quote for each subgroup, and (iv) writing synthesized statements summarizing the content and analysis of each subgroup. As a last step, we developed category headings serving as creative titles of the code groups. Table 3 presents a description of the analytic process. The first two authors were responsible for conducting an initial draft of each of the aforementioned steps in the analysis. In a step-wise process, the last author scrutinized the drafts and the team had repeated joint discussions to reach agreement before moving to the next step in the analytic process.

**Table 3:** Description of the analytic process with examples of the step-wise process.

Analytic step	Result or example
Preliminary themes were identified	Six preliminary themes were agreed upon: (i) interaction and compassion, (ii) barriers, (iii) positive effects of treatment, (iv) symptoms and discomforts, (v) challenging treatment, and (vi) improvements
Meaning units were identified and coded into code groups, which were revised and expanded upon	The analysis resulted in three agreed code groups, with three subgroups in each, that meaning units were coded into. The code group symptoms and discomforts contained the subgroups: (i) description of health conditions after experiences of war, torture, and flight, (ii) psychosocial circumstances, and (iii) change in health and wellbeing over time. The code group conditions for access to and being able to benefit from healthcare and support contained the subgroups: (i) women's position in the family and society as barriers to accessing and benefiting from healthcare and support, (ii) trust and motivation as prerequisites for benefiting from care and support, and (iii) access to, and information and knowledge about, healthcare and support to promote the patient's health and psychosocial situation. The code group experiences of treatment contained the subgroups: (i) components and routines experienced as helpful and significant in treatment, (ii) the importance of respectful treatment as a basis for creating a sense of safety, and (iii) challenges related to treatment
Subgroups were identified and meaning units were coded into these	
Condensates were produced, defined as artificial quotes representing the content of each subgroup, and illustrative quotes were identified.	Example of an excerpt of a condensate: “These events that I have been through have affected me a lot. I was very scared, especially of men, and I didn't dare to go out. I felt like I was going crazy and that there was no hope left. I had lost many people, was sad, and felt a lot of grief. I was constantly tired and without energy. It was hard to concentrate and my memory was poor.”
Syntheses were produced for each subgroup, defined as re-conceptualized comprehensive summaries of the condensates based on a perspective as a narrator	Excerpt of a synthesis: “Intense and recurring fears were described, which had a great impact on their daily lives and resulted in mental consequences such as headaches and distress. Women actively tried to avoid these stressors by adapting their behaviors. For example, one participant feared men and avoided situations when she would encounter men by herself.”
Category headings were produced, serving as brief and expressive statements of the most significant findings within each code group	Produced category headings were: (i) living and dealing with changing mental and physical manifestations in a challenging psychosocial situation, (ii) the importance of strengthening women's possibilities of overcoming structural and individual barriers to adequate health services, and (iii) supportive and respectful multi-disciplinary trauma informed care is appreciated but demanding

### 2.6 Researcher bias and reflexivity

The first two authors are health professionals (psychologist and physiotherapist) working at one of the trauma centers utilized for participant recruitment. The last author is an associate professor, a researcher, and a health professional (specialist nurse and midwife) at a Swedish university, with no clinical affiliation to the trauma centers where the recruitment and treatment took place. None of the participants were interviewed by a health professional that had been involved in her treatment. Before interviews, we informed participants about confidentiality and emphasized that the participant should feel free to criticize the care provided at the centers. Throughout the analysis, the first two authors engaged in joint discussions together with the last author to challenge their preconceptions. The last author was involved in all the steps during the analysis and took on the role to scrutinize the data from an outsider perspective.

## 3 Results

The qualitative analysis revealed various challenges and struggles encountered and dealt with by women, as they strived to improve their health and wellbeing. Significant mental and physical manifestations were recounted, amidst a demanding psychosocial context. Numerous gender-related barriers to access TIC were expressed, calling attention to the need to address structural and individual circumstances limiting women's possibilities to receive adequate treatment. When successfully coming in contact with services offering TIC, women appreciated the supportive and respectful multi-disciplinary approach applied at the centers. While they related several positive effects on their health and wellbeing to undergoing treatment, they also expressed that it had been a challenging process.

### 3.1 Living and dealing with changing mental and physical manifestations in a challenging psychosocial situation

Following their arrival in Sweden, several events and consequences had a substantial impact on the health and wellbeing of the participants. Intense and recurring fears were described, which had a great impact on their daily lives and resulted in mental consequences such as headaches and distress. Women actively tried to avoid these stressors by adapting their behaviors. For example, one participant feared men and avoided situations when she would encounter men by herself. Participants also expressed negative impacts on their mood, including sadness and hopelessness. Additional mental manifestations involved impaired concentration, memory, sleep, and recurring nightmares. These manifestations made them experience tiredness and fatigue. In addition to mental manifestations, participants also reported physical manifestations impacting their daily lives, including physical pain and bodily tensions. In some cases, the impact on their health had been so big that they previously had thoughts about not wanting to continue living.

*“I was afraid of men. It also happened here in Sweden, and we are talking about a country that is much safer. There was no reason to be afraid of all the men who came in my vicinity outside on the streets. […] In the beginning, this was something that gave me headaches, men on motorcycles, meeting men in the dark during winter.”* (Participant 1)

Participants lived in a challenging psychosocial situation, which created additional stress and affected their wellbeing negatively. Having the responsibility for their children and household, while simultaneously needing to learn a new language and securing employment, had been a substantial contributor to psychological distress. They had encountered racism at work and in the general society, but felt unable to speak up due to fear of potential consequences including deportation. Challenges in their relationship with a partner or husband were also expressed, related to previous experiences as well as new stressors in Sweden. For some, these challenges led to separation, while others reported improved relationship over time. Another substantial challenge causing significant psychological distress was the asylum claiming process, involving a fear of being deported. One participant described it as it felt like time stood still while she waited for a formal decision on her asylum claim.

*“I worked with a woman, who I worked as a personal assistant and, unfortunately I use the word that isn’t so nice, but that woman is a racist. And unfortunately, I thought no, I have to endure it because I am so scared. My fear made me get that bad vibe, bad things instead of stopping work.”* (Participant 7)

Most participants described a range of positive effects that they related to concluding the treatment at the center. Having concluded treatment, women reported less stress, anxiety, and fears. They had gained a greater understanding of their condition, how stress impacted their health, and felt less affected by previous traumatic events. Increased self-confidence, happiness, and hopefulness were also reported among participants. Their sleep quality had improved, with fewer nightmares and less fatigue. Some regained the ability to appreciate relationships, felt calmer, and were more present following the conclusion of treatment. One participant described that she was able to appreciate her surroundings in new ways following conclusion of treatment. Physical symptoms had also improved, including headaches and bodily tensions, and participants expressed an enhanced ability to manage physical manifestations. However, while most participants described health improvements over time, one woman described no health-related improvements because of the treatment.

*“When I come [to the treatment center] and talk, I feel that I can talk freely and there is someone who listens to me, and I'm fine. The first four years I lived here, it was like, I lived but only my body was here, I didn't feel anything from nature […] But now I can see the road, I feel the road now when I go there, I feel that it’s a nice road.”* (Participant 9)

### 3.2 The importance of strengthening women's possibilities of overcoming structural and individual barriers to adequate health services

Various gender-related barriers to access TIC, based of the position of women in society, were highlighted in the interviews. According to participants, women in their position are often expected to take on the main responsibility for their household and family, which can hinder access to health services providing TIC. A lack of general knowledge among women about mental disorders, available treatment, and language barriers were also mentioned as potential barriers. Participants expressed that men who are in close relationships with women may prevent them from seeking professional support, for example because of a lack of knowledge about mental health. Women exposed to domestic abuse were highlighted as especially vulnerable to not having access to needed health services, related to fears of consequences if the abuser would find out about the treatment. Children were described as a motivation to seek treatment, but offering childcare to women in need of treatment was seen as necessary in order to access treatment.

*“I am talking about women who emigrated from their countries, who have been very oppressed there or have been very badly treated by their husbands. The women who really need help here, those who have been badly treated, had a lot of problems in their home country. Even if they have arrived in Sweden, they do not know that they have the right to talk to a professional here. They may not dare to participate in such conversations.”* (Participant 6)

Internal processes within women, including emotions and thoughts related to initiating treatment, were acknowledged as barriers to health services. Negative comments from other women about mental health services, as well as doubts about their own strength to complete treatment, made them feel hesitant to seek professional support. Participants mentioned that their own motivation was a crucial component to feel able to attend treatment, and expressed that a willingness to work towards positive change had been necessary. When treatment had been initiated, their trust in health professionals grew gradually, resulting in participants feeling more comfortable over time to talk about their wellbeing and their circumstances.

*“First of all, a person, be it a woman or a man, must accept that they have depression and want to accept treatment. But if you don't want to accept and receive this kind of treatment, it won't work. You must be prepared to understand that you have depression, and want help and receive treatment. And you have to forgive yourself.”* (Participant 4)

Insufficient information and knowledge about health services were also expressed to limit women's access to TIC. Participants experienced language barriers and a lack of knowledge among health professionals in other health services, which impacted women's opportunities to access TIC at specialized centers. Fear of stigma or judgment made some women consider not seeking professional support at the centers. Another concern that could limit access was how confidentiality was handled at the trauma centers, as women worried if information disclosed during treatment would be shared with others. Interventions to enhance the access to health services providing TIC were suggested. This included improvements in information dissemination about available services, and a need for enhanced collaboration between trauma centers and other health services or other stakeholders in society.

*“[Women] may not know where to turn, what they [health services] are, who the Red Cross is, what they do. Maybe they don't dare. Some women experience violence from their husband or society, maybe they don't dare. If I go there, my husband will know. […] Knowledge is very important. If [women] don't know who [health services] are, how can they get to you? You know what I mean? You [women] have to know where to turn*.” (Participant 11)

### 3.3 Supportive and respectful multi-disciplinary trauma informed care is appreciated but demanding

Women found the multi-disciplinary treatment and services provided at the specialized centers to be beneficial for their health and wellbeing. With support from psychologists, they gained an understanding how previous trauma impacted their health, were helped to challenge their fears, and received advice how to manage their feelings. Physiotherapists taught them techniques for breathing, calmness, and to manage physical pain. Social workers supported them with handling their financial situation, family life, and the migration process. Family-based activities at the centers were appreciated as opportunities to meet others with similar experiences and situations. Participants suggested that TIC could be improved further by introducing group-based peer support, to talk about rights and share experiences with others who have similar experiences. Further, participants suggested that the centers should help women write their stories, as an opportunity for recognition. Women appreciated gaining help with referrals to other services when needed.

*“She [health professional] has taught me and shown me what to do to reduce my headaches, how to feel better physically, how to reduce my shoulder problems, how to reduce my knee pain. She has shown me, she has taught me and we practiced those exercises together. She has taught me how to deal with my anger or when I get sad or irritated.”* (Participant 3)

Participants appreciated the supportive and respectful communication encountered at the trauma centers. They emphasized the importance of a friendly, warm, kind, and patient demeanor of health professionals, who acknowledged them and listened to their experiences. Through professional support, they were encouraged to believe that positive change was possible. The trauma centers were perceived as safe spaces, where they were looked after. Some described the staff as a second family, illustrating the familiar feeling and attachment developed towards the health professionals at the centers. Women described that confidentiality had been a decisive component when choosing to initiate the treatment at the center. Thus, they highly appreciated the confidentiality and sensitivity applied at the centers, and emphasized the importance of having a confidential and safe space to talk about their experiences and situation.

*“I think that part of it is his [health professional’s] profession and knowledge, but I think the other part which is almost equally important is his personality and behavior which makes those who contact him give this trust in him and you can tell things, as you have it in the heart*.” (Participant 8)

Parts of the treatment at the centers were described as demanding, including experiencing challenges to fit sessions in a short time period and to complete assignments between sessions. The content of the sessions and assignments had been challenging, with some conversations during treatment making participants experiencing worsened symptoms and wellbeing, particularly during early phases of the treatment. Participants reported that the treatment had triggered worsened sleep, more nightmares, intrusive memories, fatigue, increased tensions, and physical pain. Talking about their memories had been challenging, with some crying during the conversations. Because of feelings associated with the conversations, some participants had worried about the content of upcoming treatment sessions. One woman described feeling deeply depressed at the beginning of treatment, likening the conversations with health professionals to prison interrogations. The experienced escalation of symptoms because of the treatment had such an impact on some women that they considered terminating their treatment, and some described that the increased symptoms persisted over several days. On the other hand, participants described that the treatment became gradually less burdensome over time, making them feel encouraged to continue on. Some refused to participate in group-based activities, because they found it difficult to attend sessions together with other women.

*“At [the trauma center] when I got this treatment, the last treatment I got. I thought like this, at the beginning, that it was a lot of work. You can feel, like, I can't stand it. I feel like I'm in a prison and someone is sitting in front of me asking questions all the time and they force me to answer and take care of it. […] I was completely exhausted. I couldn't, I felt it all coming at once. I saw in front of me my nightmares and this event and everything. Those were the worst days. But then it got better and better.”* (Participant 4)

## 4 Discussion

This study explored the experiences among migrant women who have concluded trauma-informed treatment at specialized centers. The findings revealed mental and physical manifestations while living in a challenging psychosocial situation and needing to overcome barriers to health services. Although demanding to undergo treatment, women appreciated various benefits and valued the compassionate care provided at the specialized centers. The findings confirm previous reports highlighting the structural disadvantages, oppression, and violence enacted against forced migrant women [21,25]. This study addresses and build on previous research about a population of women that is continuously being underrepresented in research [21,26].

To address the impactful health-related consequences highlighted in our and other studies [5,[7], [8], [9]], the need for accessible evidence-based TIC for women who are forced migrants is undeniable. Migrant women experience unmet health needs and encounter barriers when trying to access health services in the host country [9,27]. Echoing previous research about barriers to health services among women exposed to intimate partner violence [28] and forced migrants [29], we identified gender-related barriers to access specialized services. These findings contribute to the knowledge gap concerning migrants' own views on barriers to health services [9]. One of the barriers emphasized by our participants was gender-based intimate partner violence, which is a major public health issue and is a prevalent problem in society [30,31]. Society has an important responsibility to reach women exposed to domestic violence, to provide adequate support. Our participants suggested improved dissemination of information and more collaboration between health services and other actors, to enhance women's access to treatment services. Indeed, previous research call attention to the fragmented health services for women who are forced migrants, involving insufficient information, lack of sensitivity towards the unique needs of women, and an unavailability of services [32]. Outreach efforts through the provision of accessible information in collaboration with communities have been suggested as a way to identify those in need of specialized services [20,33], but more research is needed. Specialized centers providing TIC should consider how to further enhance the potential to reach forced migrants in need of support.

Research show that clients in TIC emphasize the importance of appropriate interpersonal communication and safe spaces [34], which was confirmed by the participants in our study. Promoting emotional wellbeing through trust, respect, hope, and empowerment is paramount when supporting clients with traumatic experiences [35]. Indeed, guidelines emphasize the importance of ensuring that clients feel emotionally, physically, and socially safe, to reduce the risk of inducing anxiety and re-traumatization [36,37]. When providing TIC for women, approaches focusing on empowerment and resilience has the potential to facilitate adaptation and recovery [20]. However, ethnic minority and migrant women encounter discrimination and exclusion when trying to access specialist services [33]. Despite the need for effective structures supporting traumatized individuals, there is a lack of empirical research investigating specific interventions targeting women [26,38]. Social support is acknowledged as a key principle in TIC [17,37] and has been highlighted as a potentially valuable resource for coping and resilience among refugee women [39]. Research suggests that group-based sessions aiming to empower women who are victims of intimate partner violence can help improve quality of life and alleviate health burdens [40]. However, there is a need for more research to draw conclusions about the potential utilization and impact of TIC services offering social support for these women.

An important finding is the adverse events of treatment described by some participants. There is a lack of reporting of adverse events of psychotherapy [41], with the need for more rigorous and systematic procedures to identify unwanted effects [42]. Talking about experiences of previous traumatic events involves a risk of inducing psychological distress [43], which was described by our participants. Participants referred to the treatment as demanding and burdensome, calling attention to the need for sufficient motivation and strength to conclude the treatment. Some described that they deliberated to prematurely terminate the treatment due to the experienced escalation of symptoms. Premature discontinuation leading to dropout of therapy is highlighted in research as a major problem [44,45]. In this study, we only included women who had concluded their treatment. Thus, the findings do not reflect the experiences of those who dropped out during treatment. This calls attention to the need for more research that explores considerations needed to increase adherence among women in this target population.

There are methodological limitations that need to be considered when interpreting the findings of this exploratory qualitative study. While we recruited participants from five centers geographically dispersed throughout Sweden, no purposeful selection was performed. We utilized a reverse chronological sampling procedure to recruit participants, which allowed us to interview participants who recently underwent treatment. On the other hand, the sampling procedure could implicate that women with positive impressions of treatment were interviewed. Thus, it is possible that we did not achieve a variation in experiences within the sample. However, the sample was varied in regard to age, country of birth, legal status, and educational level. The data was collected and analyzed by two health professionals employed at one of the five centers used for recruitment. This involves a risk of participant non-disclosure during data collection and interpreter bias. However, none of the interviewers had been involved in the participant's treatment and the last author was also involved in all steps of the analysis in the role of scrutinizing researcher. Nevertheless, we cannot dismiss this risk of bias and the findings needs to be interpreted with this in mind. The interviews were carried out either in face-to-face setting or remotely via video conferencing/telephone. We believe that both methods generated rich data. Furthermore, we did not collect any quantitative measures on health-related variables, and thus, we cannot make any assumptions about the clinical levels of health burdens among participants. A qualitative design was used to explore the experiences of women in an inductive approach, and we interviewed a total of eleven women. Sample size in qualitative research depends on the richness and variation of the data collected, as the goal is to elucidate nuances and to contribute to new understandings [24]. While we argue that our study achieves this, we encourage more qualitative research that explores experiences among women who have undergone trauma-informed treatment in other contexts and with diverse samples.

## 5 Conclusions

Women who have undergone treatment at specialized centers applying TIC tell stories illustrating a range of mental and physical manifestations, which affect their daily lives while living in a challenging psychosocial situation. Various gender-related structural and individual barriers hinder women access to TIC at specialized centers. When participating in treatment, women experience a range of health-related benefits. A respectful and supportive demeanor of health professionals is important and appreciated by women. On the other hand, women describe the treatment as demanding, requiring energy and determination. Health professionals and stakeholders at specialized centers providing TIC for forced migrants need to ensure that their services are accessible and culturally sensitive towards the unique needs of women. Outreach efforts with efficient dissemination of information about the available support have the potential to break down some of the barriers hindering women to access treatment. Women suggest group-based peer support activities as additional interventions that could enhance the support further, which need evaluation in future research.

## Data availability statement

Due to ethical reasons related to confidentiality, the data is not publicly available.

## Ethics statement

The study was approved by the Swedish Ethical Review Authority (approval number 2022-03636-01). All participants provided oral and written informed consent before data collection commenced. No health professional involved in their previous treatment had contact with them to inform about the study, nor to collect any data. The data was handled confidentially and digitally stored in locked storage at one of the trauma centers where the study was based.

## Additional information

No additional information is available for this paper.

## Funding

This study was funded by the Swedish Red Cross, 10.13039/501100009780Region Skåne, and Skåne Association of Local Authorities (Swedish: “Kommunförbundet”). The funders had no role in the study design, in the collection, analysis and interpretation of data, the writing of articles, or the decision to submit for publication.

## CRediT authorship contribution statement

**Linda Jolof:** Writing – original draft, Project administration, Formal analysis, Data curation, Conceptualization. **Patricia Rocca:** Writing – original draft, Formal analysis, Data curation, Conceptualization. **Tommy Carlsson:** Formal analysis, Conceptualization, Supervision, Writing – original draft.

## Declaration of competing interest

The authors declare the following financial interests/personal relationships which may be considered as potential competing interests: Linda Jolof reports financial support was provided by Region Skåne. Patricia Rocca reports financial support was provided by The Red Cross Treatment Center for persons affected by war and torture. Linda Jolof reports financial support was provided by Skåne Association of Local Authorities. Patricia Rocca reports financial support was provided by Skåne Association of Local Authorities. Linda Jolof and Patricia Rocca reports a relationship with The Red Cross Treatment Center for persons affected by war and torture that includes: employment. Linda Jolof and Patricia Rocca declare no other competing interests. The last author declares no competing interests.
